# Low Cost Plastic Optical Fiber Pressure Sensor Embedded in Mattress for Vital Signal Monitoring

**DOI:** 10.3390/s17122900

**Published:** 2017-12-13

**Authors:** Demetrio Sartiano, Salvador Sales

**Affiliations:** Institute of Telecommunications and Multimedia Applications (iTEAM), Universitat Politècnica de València, Camino de Vera s/n, 46022 Valencia, Spain; ssales@dcom.upv.es

**Keywords:** plastic fiber sensor, mattress sensors, sleep monitoring, non-invasive respiration monitoring

## Abstract

The aim of this paper is to report the design of a low-cost plastic optical fiber (POF) pressure sensor, embedded in a mattress. We report the design of a multipoint sensor, a cheap alternative to the most common fiber sensors. The sensor is implemented using Arduino board, standard LEDs for optical communication in POF (λ = 645 nm) and a silicon light sensor. The Super ESKA^®^ plastic fibers were used to implement the fiber intensity sensor, arranged in a 4 × 4 matrix. During the breathing cycles, the force transmitted from the lungs to the thorax is in the order of tens of Newtons, and the respiration rate is of one breath every 2–5 s (0.2–0.5 Hz). The sensor has a resolution of force applied on a single point of 2.2–4.5%/N on the normalized voltage output, and a bandwidth of 10 Hz, it is then suitable to monitor the respiration movements. Another issue to be addressed is the presence of hysteresis over load cycles. The sensor was loaded cyclically to estimate the drift of the system, and the hysteresis was found to be negligible.

## 1. Introduction

The development of optical fiber sensors started during the 1970s; since these years, the research was mostly focused on glass optical fibers. Research leads to fiber sensors for strain and temperature, and the development of distributed sensors through the inscription of gratings or exploitation of nonlinear optical effects. These sensors have a wide range of applications from structure health monitoring [1] to wearable for medical purposes [2]. Nowadays, in wearable technology, electronic sensors are the most employed; new textile, smart shirts, and shoe-based sensors were developed to monitor: body fluids during exercise [3] and physiological parameters [4]; posture allocations and activities [5]; stress monitoring [6]; and hand gesture and activity recognition [7]. Optical fiber sensors offer an alternative that is lightweight and immune to electromagnetic field, making them useful to monitor patients during diagnostic procedures in presence of high electromagnetic field, such as during magnetic resonance imaging [8].

Plastic optical fiber applications are growing in wearable sensors field, since their mechanical properties make them completely embeddable in textile; some studies proved that they can be processed in high volume production, manufactured and integrated as either weft or warp in the weave [9]. Many research works present plastic optical fiber (POF) sensors based on intensity variations due to bending [10], or exploiting the macro-bends properties for sensing liquid-level [11], force [12] and temperature [13]. These approaches permit implementing low-cost sensors that can be interrogated using a simple optoelectronic scheme (LED–fiber–photodetector). In general, both glass and plastic optical fibers permit developing low cost alternatives to already existing sensors, for example to monitor joint movements [14] and spinal posture [15], as well as sensors for medical application, e.g., sleep apnea [16] and respiration monitoring [2]. In [16], it is presented a plastic optical fiber sensor embedded in a mattress for monitoring sleep apneas. Sensors in mattress permit to monitor the patient without requiring the user to wear anything, there are some commercial devices capable not only of diagnosing nocturnal apneas [17,18], but also of monitoring sleep phases [19].

In this paper, we report the design of a multipoint sensor developed with standard plastic optical fibers and embedded in a mattress. Our sensor is a cheap alternative: an intensity based sensor implemented with an Arduino and standard electronic components for plastic fiber application (e.g., data link). Standard plastic fibers were chosen for the sensor and they were made sensitive to pressure with a simple method that can be reproduced for high volume production. The performance in terms of resolution and the software that process the light intensity data allow monitoring movements and respiration. All code for the data processing was implemented in the ATmega2560 microcontroller (Atmel, San Jose, CA, USA) of the Arduino; a computer is needed only to plot the serial data sent from the board. Adding a memory to store all the data or a wireless/Bluetooth module to the board we can obtain a portable, stand-alone sensor.

The paper is divided into the following sections: Section 2 presents the principle of operation of the sensor. Section 3 describes the practical sensor implementation. In Section 3, the measurements carried out to test the sensor performance in terms of linearity, frequency response and time drift under repetitive load cycles are presented. In Section 4, the conclusions are presented.

## 2. Principle of Operation

**Figure 1:**
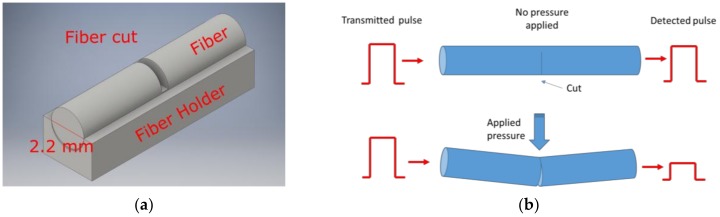
(**a**) 3D model of the plastic holder used to cut the fibers. The hemicylindrical groove has the same diameter as the plastic fiber to limit the penetration of the blade into the fiber and obtain similar cuts of approximately 1.1 mm (half of the fiber diameter) depth. (**b**) Working principle of a single point of the fiber sensor.

The sensor is based on the intensity modulation of light in plastic optical fibers (POF). The fibers are the industrial plastic ones manufactured by Mitsubishi: the Super ESKA^®^ (Mitsubishi Rayon Co. LTD., Tokyo, Japan). The fibers have a core of polymethyl-methacrylate of 980 µm and a cladding of fluorinated polymer with a diameter of 1000 µm. We use the type with a polyethylene jacket of 2.2 mm. The POFs have been sensitized to pressure, cutting part of the jacket, the cladding and the core (Figure 1a). A fiber holder with a hemicylindrical groove with the same diameter of the fiber was used together with a razor blade to obtain cuts with approximately the same depth. As described in Figure 1b, any applied pressure on the cut point changes the light intensity at the fiber end.

**Figure 2:**
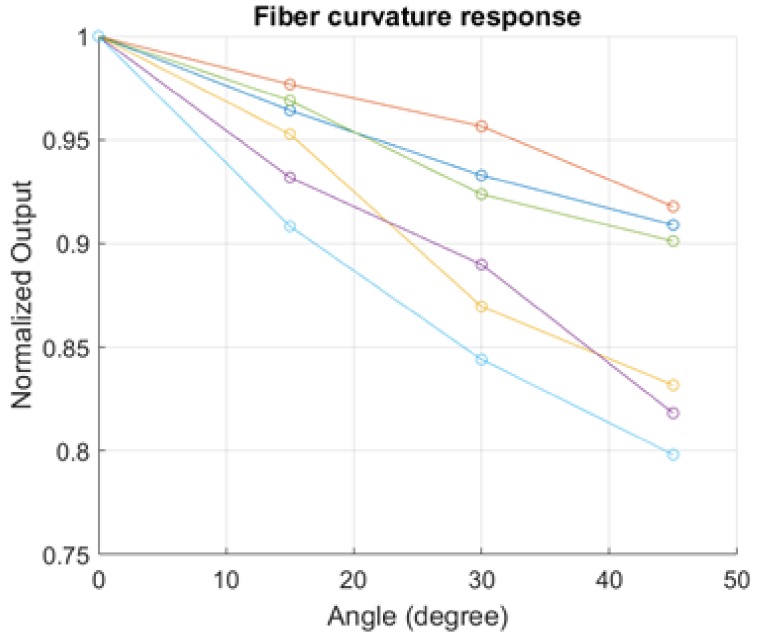
Normalized response to curvature of six sensitive points.

Different techniques were presented to make “sensitive” POFs to bending: abrading part of the cladding [20,21], and using a solvent that can attack the cladding without damaging the core [22]. We focus on finding a technique that can be reproduced in high-volume production. Moreover, after every cut, the fibers were mounted on a goniometer for curvature response measurement, and interrogated in transmission; since a pressure applied bends the fiber with a certain angle, the curvature response gives a first estimation of the pressure response of the cut fibers. Figure 2 shows the response of six different cut fibers to curvature in terms of average normalized light power measured at the fiber end, and Table 1 reports a table with the averages of the values obtained and the standard deviations, for different angles of curvature.

**Figure 3:**
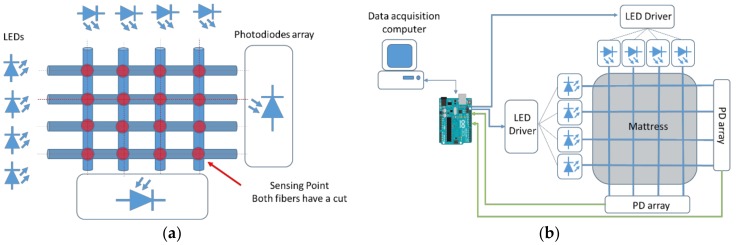
(**a**) Schematic representation of the fiber matrix; and (**b**) sensor implementation: the arrows represent the communication between the computer, the board and the electronic components of the interrogation circuit.

The fibers were then arranged in a matrix with the cuts positioned in the crossing points (Figure 3a) and embedded in a mattress cover sample. The computer acquires the data from the board, that is the actual core of the sensor, since it controls the current (light intensity) in the LEDs and converts the voltage values that are serially outputted from the photodetectors array (Figure 3b).

The position of the pressure applied can be retrieved from the simultaneous interrogation of the fibers in the matrix: as is described in detail in Section 3, each fiber is associated with an average light intensity, obtained processing the light data at fiber end. An intensity variation for two different fibers is considered as a pressure applied on the crossing point between the latter; monitoring the output power of all the fibers, we can reconstruct the pressure matrix.

## 3. Sensor Implementation

**Figure 4:**
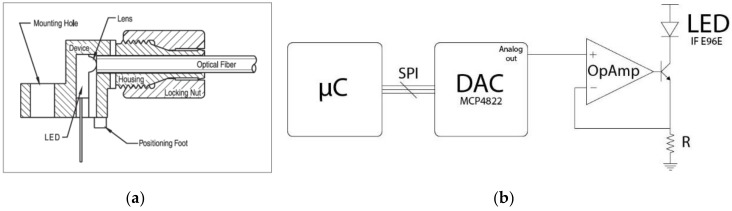
(**a**) Cross section of the LED used to inject light in the plastic fiber; and (**b**) schematic representation of the current driver circuit.

The LED used to inject the light in the fiber is the IF E96E (Industrial Fiber Optics, Tempe, AZ, USA), a low-cost, high-speed, visible red LED housed in a “connector-less” style plastic fiber optic package (Figure 4a). The output spectrum is produced by an AlGaInP die which peaks at 645 nm, one of the optical transmission windows for POFs. The LEDs are driven with constant current using the driver circuit shown in Figure 4b. It was implemented with a 12-bit digital-to-analog converter (DAC) (MCP4822 (Microchip, Chandler, AZ, USA)), a general purpose operational amplifier (LM358 (Texas Instrument, Dallas, TX, USA)), an NPN silicon BJT transistor (2N2222A (STMicroelectronics, Geneva, Switzerland)) and one resistor. The microcontroller communicates through the SPI interface with the DAC that fixed the voltage at the (+) pin of the operational amplifier. The negative feedback of the system implemented with an OpAmp, a BJT transistor and a resistor “copies” the voltage of the (+) pin to the (−). In this way, we can fix the current of the LED since I_LED_ ≈ V_DAC_/R. The light is injected in the form of a pulse with constant duration. The duration of the pulses was chosen to maximize the full-scale range of the system. The lower pulse width is limited by the maximum operating frequency of the analog stage and by the LED bandwidth (maximum operation frequency 50 Mbps); the upper limit was chosen in order to not saturate the photodiodes. The duration was fixed to 50 µs.

**Figure 5:**
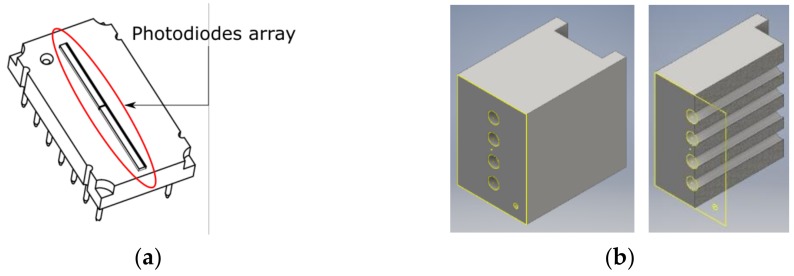
(**a**) Photodetectors array TSL1402R; and (**b**) 3D model of the fiber holder, entire piece (left) and cross-section (right).

The photodetectors array is the TSL1402R (ams AG, Styria, Austria) (Figure 5a), a linear array of 256 photodiodes. A plastic component was specifically designed, and 3D printed to bottom couple the fibers ends to the light sensor (Figure 5b).

The customized coupler holds the end of the fibers in front of the sensitive area of the light sensor and insulates the photodiodes from the ambient light that could influence the measurement. Light energy impinging on a pixel generates a photocurrent, which is then integrated by the active integration circuitry associated with that pixel. The amount of charge accumulated at each pixel is directly proportional to the light intensity on that pixel and the integration time. The charge stored is sequentially connected to a charge-coupled output amplifier that generates a voltage on analog output. The output voltage of the sensor is converted to a digital value through the build-in analog-to-digital converter (ADC) of the Arduino board. The digital output of the 10-bit ADC is directly proportional to the optical power irradiated at the end of the fiber, according to the formula:

(1) Digital output = (P×Re×10n bit)/VDD

where P is the incident light energy, R_e_ is the responsivity of the photodetectors, n bit is the number of bits of the ADC and V_DD_ is the high analog voltage level.

**Figure 6:**
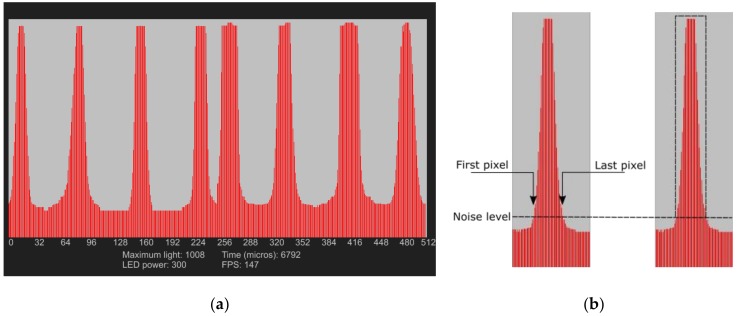
(**a**) Plot of the light level detected by the two arrays of photodetectors, the eight peaks are the light outputs of the eight fibers bottom coupled to the photodetectors array; and (**b**) light signal post-processing: the software individuates and store the first and last pixel position that detect a light power above a certain threshold (noise level), any variation of light power in this area will be interpreted as a pressure applied in one of the sensitive points of the fiber.

The user interface was programmed in Java to visualize the light energy impinging on the pixels of the photodiodes array. The plot in Figure 6a shows the output of the two photodetectors arrays. The x-axis represents the pixel numbers, and the y-axis the output of the ADC. The red peaks are the light detected at the fiber ends; the software implemented in the microcontroller identifies the position of every single fiber coupled to the photodetectors array, and it averages the optical power within the peaks area. Specifically, fixing a noise level, it determines the first and last pixel where a fiber end is coupled and operates an averaging of the voltage signals; the principle is shown in Figure 6b. This permits obtaining a measurement less sensitive to the optical power fluctuation due to the electronic noise and to the non-perfect fiber-photodetectors array coupling, which could directly influence the voltage level at the input of the ADC. Moreover, for small pressure, the light power losses are more evident in the extreme pixels of the fiber end area, thus averaging is needed since monitoring only the maximum peaks would not make it possible to also detect these small signal variations.

## 4. Measurements and Discussion

### 4.1. Force Measurements

One static and two dynamic tests were carried out to analyze the performance of the sensor:

1. The first static test was made placing weights in a range 0–500 g (0–4.9 N) with step of 100 g (force = 0.98 N) on the sensitive points of the matrix.
2. The second test is a dynamic test: a force step stimulus was applied on a point of the mattress sensor. From the time response data, the frequency response was obtained operating the Fourier transform.
3. The third is a cyclic loading test.

For the static tests, the force was applied on four different points, and the charge cycles were repeated three times. Since the idea is to use this sensor for the dynamic monitoring of vital signs, as for example the respiration rate (0.2–0.5 Hz), we would like to obtain a bandwidth of 10 Hz. The load cycle test was carried out to determine the short time drift or hysteresis of the sensor, which can be caused by the plastic deformation of the fibers. Weights for scale placed into a cylindrical weighing pan were used to apply the load on the sensitive points of the matrix both for static and dynamic measurements.

### 4.2. Static Measurement

**Figure 7:**
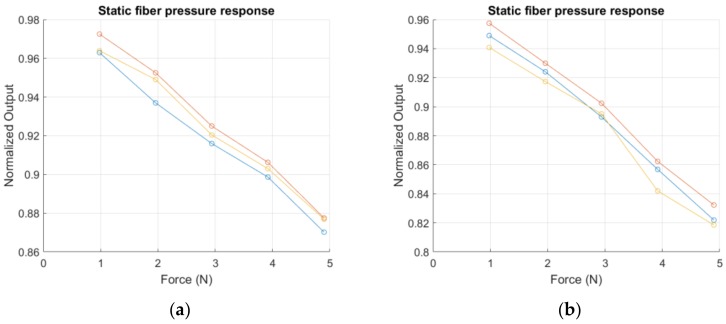

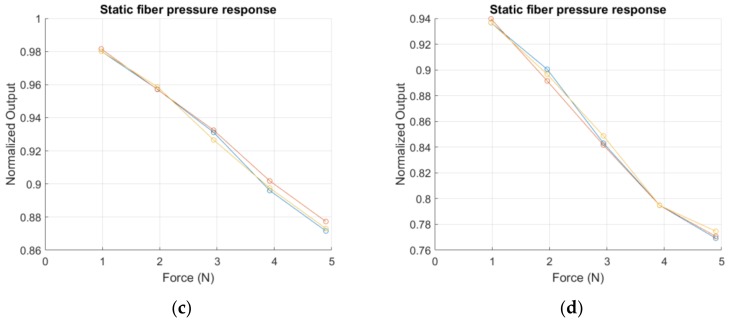
The plots represent the response of four different points to an increasing static load. The measurements were repeated three times and plotted with different colors. The linearity for the single measurement was evaluated, the results are reported in Table 2: (**a**,**c**) side points tested on the cover sample; (**b**,**d**) central points tested on the cover sample.

**Figure 8:**
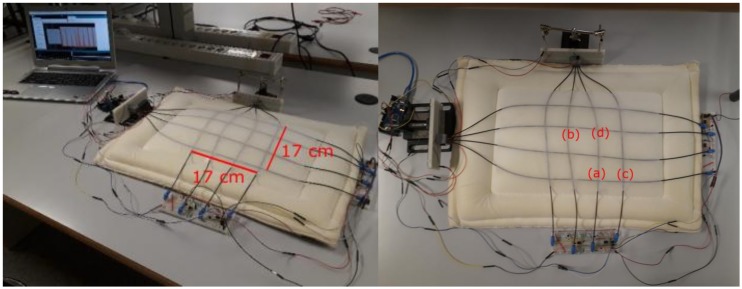
(**a**) Experimental set-up; and (**b**) mattress cover with the integrated POFs, the points tested to measure the static response are circled and numbered.

The charge cycles, for obtaining the static response, were repeated three times for every single sensitive point to verify the repeatability of the measurement. The step and the range of the measurement were chosen considering the pressure changes in the lungs during the breathing cycle, which are partially transmitted to the thorax. The pressure changes are in the order of 5 cm H_2_O (≈490 Pa); considering the average thorax area for an adult [23], an applied force of about 50 N is obtained. As commented before, the pressure is only partially transmitted to the thorax expansion movement, so we assume that a resolution of 1 N could be a reasonable resolution to detect the force applied during respiration. Figure 7a–d represents the normalized responses of four single points of the matrix placing weights in a range of 0–500 g (0–4.9 N) with step of 100 g (force = 0.98 N); the measurement was repeated three times for every point, the repetitions are represented in the figures with different colors. The measurements show good linear responses: in Table 2 are reported the linear regression results (alpha = first degree term; beta = constant term and R^2^ value). The resolutions obtained are slightly different for the four points, due to the different location of the tested points on the mattress cover sample. Points 7b and 7d are in the center of the cover, so the weight apply a perpendicular pressure on these sensitive points (resolution of 3–4%/N on the normalized voltage output), while Points 7a and 7c are on the side of the mattress sample (resolution of 2%/N on the normalized voltage output). The experimental set-up is represented in Figure 8a; in Figure 8b is presented from a different perspective of the set-up, where the points tested for the static response are highlighted.

### 4.3. Frequency Response

**Figure 9:**
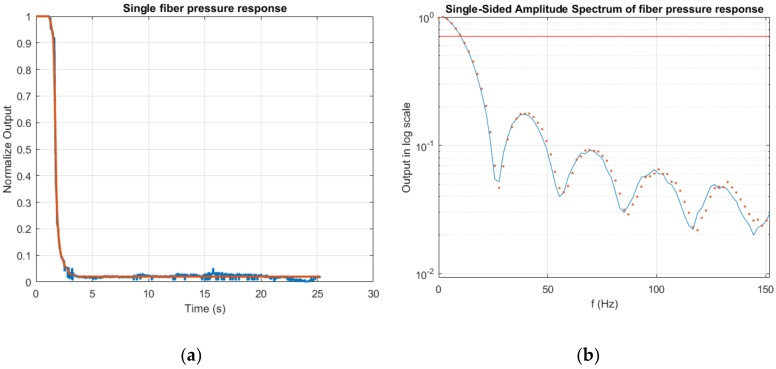
(**a**) Time response of a fiber, after a pressure is applied: in blue is reported the response measured and in red the theoretical response calculated considering the frequency response of the electronics. (**b**) Frequency response retrieved from the time response data calculating the derivative of the Fourier transform. In red is represented the −3 dB line, the theoretical impulse response of the system is plotted with red dots.

For the dynamic measurement, we want to test the response of the sensor for stimulus with frequencies lower than 10 Hz. Usually, to study the frequency behavior of a fiber sensor, piezo fiber stretchers are used [24]; since we want to study the response for very low frequencies, we carried out an analytical study of the time response data. The experimental result was compared to a theoretical result, computed considering the frequency response of the electronic light sensor and converter circuits (photodiode array-ADC). In Figure 9a in blue is represented the experimental response of our fiber sensor when a step like pressure stimulus is applied, while in red is represented the simulated response for the same light attenuation obtained in the practical experiment.

If we assume that the response of the sensor is linear for the load step applied, it is possible to obtain the frequency response of the sensor calculating the derivative of the Fourier transform of the time step response data. The result is shown in Figure 9b (blue line). On the frequency plot is reported the −3 dB line in red, as it can be observed that the response is >−3 dB for frequencies <10 Hz. This response proves that the sensor is feasible for detecting the respiration movement of a user in contact with the mattress sensor. As for the time response, the computed frequency response was compared to the ideal impulse response of our electronic system; the theoretical curve is reported in Figure 9b plotted with red dots.

### 4.4. Loading Cycles

**Figure 10:**
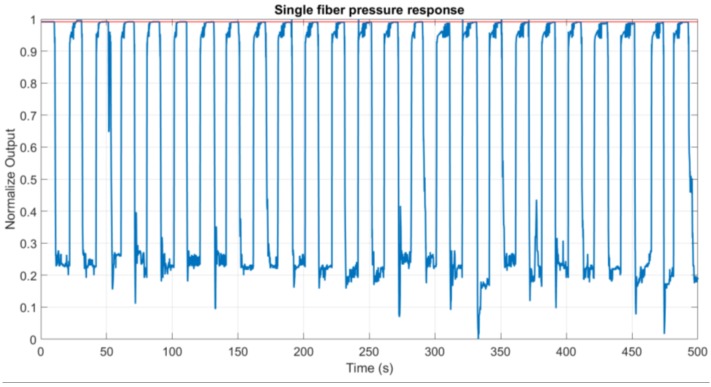
Cyclic loading test to verify the presence of a drift in the response, in red is represented the initial zero-pressure base line.

One of the problems to address for plastic fibers is that often plastic deformation can occur, so the sensitive point cannot recover completely after a pressure is applied, adding a constant offset that could adversely affect the sensor performance. Therefore, it is necessary to run a cyclic pressure test to verify that the zero-pressure level does not change significantly. In Figure 10, in red, is reported the zero line (when no pressure is applied) and the response for a cyclic loading (in blue). The measurement demonstrates that substantially the zero-pressure level does not change after 25 short time load cycles with a force of 20 N.

## 5. Conclusions

We reported the design and the practical implementation of a fiber sensor integrated into a mattress for non-invasive monitoring of movements and respiration. The sensor was implemented with standard electronic components for plastic fiber optical communication, an Arduino, and industrial POFs, which were prepared to detect pressure, using a simple and repeatable method that could be easily reproduced in a large-scale production process. The performance obtained demonstrates the feasibility of the sensor for detecting movements and respiration. Static measurements show good linearity (R^2^ values > 97.40%), and a resolution between 2.2%/N and 4.5%/N. The sensor has a response >−3 dB for frequency lower than 10 Hz and no drift due to loading cycles. The results obtained make the sensor feasible for sleep apnea detection and in general for non-invasive cardio pulmonary monitoring. After this first implementation, the work will be focused on enhancing the data processing to suppress long terms instabilities and hysteresis and implement machine learning techniques to investigate the detection of respiration anomalies on real patients. For the detection of such variations in the respiration movements, a system with an ADC with higher resolution has to be considered, as well as a reference branch for monitoring the LED intensity stability for long measurement. For this first implementation, the instability in the light intensity of the LED was not addressed since, even for long measurement, we did not observe any variations in the output light intensity detected.

## Figures and Tables

**Table 1:** Average normalized responses and standard deviations at different curvature angles.

Angle	Normalized Response ± Standard Deviation
15°	0.9452 ± 0.0251
30°	0.8920 ± 0.0370
45°	0.8516 ± 0.0503

**Table 2:** Results of the linear regression for the loading static test. The alpha is the first-degree term of the linear regression, the beta is the constant term. The R^2^ values are reported in the third columns.

Sensitive Point	Repetition	Alpha	Beta	R^2^	Sensitive Point	Repetition	Alpha	Beta	R^2^
(a)	1	−0.02283	0.98405	99.40%	(b)	1	−0.03279	0.98531	99.50%
	2	−0.02411	0.99762	99.60%		2	−0.03244	0.99224	99.50%
	3	−0.02246	0.98868	99.20%		3	−0.03263	0.97864	97.40%
(c)	1	−0.02840	1.01070	99.50%	(d)	1	−0.04494	0.98100	98.70%
	2	−0.02692	1.00920	99.80%		2	−0.04433	0.97798	98.70%
	3	−0.02809	1.00980	99.70%		3	−0.04346	0.97809	98.50%
